# Evaluation of Bioelectrical Activity of Pelvic Floor Muscles and Synergistic Muscles Depending on Orientation of Pelvis in Menopausal Women with Symptoms of Stress Urinary Incontinence: A Preliminary Observational Study

**DOI:** 10.1155/2014/274938

**Published:** 2014-02-19

**Authors:** Tomasz Halski, Lucyna Słupska, Robert Dymarek, Janusz Bartnicki, Urszula Halska, Agata Król, Małgorzata Paprocka-Borowicz, Janusz Dembowski, Romuald Zdrojowy, Kuba Ptaszkowski

**Affiliations:** ^1^Department of Physiotherapy, Public Higher Medical Professional School in Opole, Katowicka 68, 45-060 Opole, Poland; ^2^Department of Clinical Biomechanics and Physiotherapy in Motor System Disorders, Faculty of Health Science, Wroclaw Medical University, Grunwaldzka 2, 50-355 Wroclaw, Poland; ^3^Department of Nervous System Diseases, Faculty of Health Science, Wroclaw Medical University, K. Bartla 5, 51-618 Wroclaw, Poland; ^4^Department of Obstetrics, Faculty of Health Science, Wroclaw Medical University, K. Bartla 5, 51-618 Wroclaw, Poland; ^5^Department of Obstetrics and Gynecology, Health Center Bitterfeld/Wolfen gGmbH, Friedrich-Ludwig-Jahn-Straße 2, 06749 Bitterfeld-Wolfen, Germany; ^6^Department of Physiotherapy Basics, Academy of Physical Education in Katowice, Mikolowska 72, 40-065 Katowice, Poland; ^7^Department and Clinic of Urology, Faculty of Postgraduate Medical Training, Wroclaw Medical University, Borowska 213, 50-556 Wroclaw, Poland

## Abstract

*Objectives*. Evaluation of resting and functional bioelectrical activity of the pelvic floor muscles (PFM) and the synergistic muscles, depending on the orientation of the pelvis, in anterior (P1) and posterior (P2) pelvic tilt. *Design*. Preliminary, prospective observational study. *Setting*. Department and Clinic of Urology, University Hospital in Wroclaw, Poland. *Participants*. Thirty-two menopausal and postmenopausal women with stress urinary incontinence were recruited. Based on inclusion and exclusion criteria, sixteen women aged 55 to 70 years were enrolled in the study. *Primary Outcome Measures*. Evaluation of resting and functional bioelectrical activity of the pelvic floor muscles by electromyography (sEMG) and vaginal probe. *Secondary Outcome Measures*. Evaluation of activity of the synergistic muscles by sEMG and surface electrodes. *Results*. No significant differences between orientations P1 and P2 were found in functional and resting sEMG activity of the PFM. During resting and functional PFM activity, higher electrical activity in P2 than in P1 has been recorded in some of the synergistic muscles. *Conclusions*. This preliminary study does not provide initial evidence that pelvic tilt influences PFM activation. Although different activity of synergistic muscles occurs in various orientations of the pelvic tilt, it does not have to affect the sEMG activity of the PFM.

## 1. Background

Normal ageing of the reproductive system in women can be divided into periods as follows: reproductive (premenopausal), menopausal transition (perimenopausal), and postmenopausal. In the perimenopausal period characteristic symptoms of menopause begin to appear [1–4]. Many studies [1–6] of prevalence of menopause have reported symptoms of menstruation dysfunctions, symptoms of menopausal syndrome (hot flushes, profuse sweating, sleep disorders, irritability, depression, dizziness, headache, articular and muscular pain, and general weakness), and libido dysfunctions. Menopause may also be an etiological factor in the development or progress of urinary incontinence (UI) [1, 7–10]. In the postmenopausal period, stress urinary incontinence (SUI) appears often. It is probably connected with hormonal disturbances leading to muscle and fascial flaccidity and their decreased tone [6, 7]. Prevalence of UI in women ranges from 5% to 62% [11–18] and the incidence of UI changes with age [9]. Studies of Minassian et al. [9] show that the median of prevalence of all types of UI in women clearly increases in the 35–44 age group and is reaching about 30%. Maximum occurrence of the UI is in the 45–54 age group and is about 70% and in the age groups: 35–44 (nearly 60%), 55–64 (more than 60%) and 65–74 (about 50%). According to other studies [7–11], the estimated percentage of women suffering from UI is up to 73% during the peri- and postmenopausal periods.

To reduce the occurrence of the UI and in particular the incidence of SUI, some form of effective therapy should be applied. It is well known that a form of conservative treatment is recommended as the first-line procedure but such still needs to be improved [19–26]. The physiotherapeutic treatment of SUI is mainly focused on achieving increased resting and functional activity of the pelvic floor muscles (PFM) [21, 27–33]. Activation of isolated contractions of the PFM as well as the activation of synergistic muscles should increase the effectiveness of the treatment. In the literature [21, 32–36], muscles considered as important in the treatment of SUI are gluteus maximus; medial femoral; rectus abdominis; and oblique, external, and internal abdominal muscles. These trunk and hip muscles that attach to the pelvis or sacrum have an influence on the motion of the pelvis as a whole. Pelvic orientation depends on the contraction or extension of these muscles. Increased anterior pelvic tilt can result from weak hamstrings or abdominals, hypertonicity of the lumbar extensors or hip flexors, or contractures of the rectus femoris. While the posterior pelvic tilt could be a result of shortened hamstrings, hypertonic abdominals, weakened lumbar flexors or hip extensors [37–42]. Additionally, these muscles are functionally and morphologically connected with the pelvis and indirectly with the PFM [36, 38, 40, 43]. Taking into account these interdependencies, the authors decided to assess the PFM and synergistic muscle function in various positions of the pelvis. To evaluate bioelectrical activity of these muscles surface electromyography (sEMG) was used [44].

## 2. Objective and Hypothesis

The primary aim is the evaluation of resting and functional bioelectrical activity of the PFM, depending on the orientation of the pelvis, in forward and backward inclination. We assume that higher bioelectrical activity of the PFM will be observed in the posterior pelvic tilt than in the anterior pelvic tilt.

The secondary aim is the evaluation of the activity of synergistic muscles at different orientations of the pelvis. Higher muscle activity is expected in the posterior pelvic tilt. The higher activity of these synergistic muscles can lead to an increase of PFM activity.

## 3. Material and Method

### 3.1. Design

Preliminary, prospective, cross-sectional observational study evaluating resting and functional PFM activity depending on pelvic orientation in menopausal and postmenopausal women with SUI.

### 3.2. Participants

Thirty-two menopausal and postmenopausal women with SUI were recruited from volunteers and patients at the Department and Clinic of Urology, University Hospital in Wroclaw, Poland. The study was approved by the Bioethics Committee of the Wroclaw Medical University (KB-611/2012) and was registered at the Australian New Zealand Clinical Trials Registry (ACTRN12613001144707). The project was funded by the National Science Centre allocated on the basis of the decision number DEC-2011/03/N/NZ7/00505.

Included were women of ages 55 to 70 years with good general well-being on the day of the examination and who were able to contract the PFM correctly. All patients reported symptoms of menopause (hot flushes, sweating, heart discomfort, sleep problems, depressive mood, irritability, anxiety, physical and mental exhaustion, sexual problems, bladder problems, dryness of vagina, and joint and muscular discomfort), which were verified by the Menopause Rating Scale (MRS) [45] and symptoms of SUI (especially leaks when coughing or sneezing, leaks during physical activity/exercising, or leaks when lifting heavy objects) which were evaluated by the International Consultation on Incontinence Questionnaire-Short Form (ICIQ-SF) [46]. All included women had a history of stress urinary incontinence and gave written consent to participation in the study.

Subjects were excluded if they could not comprehend Polish instructions; had a previous history of gynaecological and abdominal surgery; had a malaise on the examination day (the participants of the study were not able to perform the test procedures); had a neurological condition; had contraindications to measurements such as infection, menstruation, and allergy to nickel; had a previous history of injuries within the pelvis, hip joint, or spine; or had a vaginal prolapse.

### 3.3. Experimental Protocol

**Figure 1:**
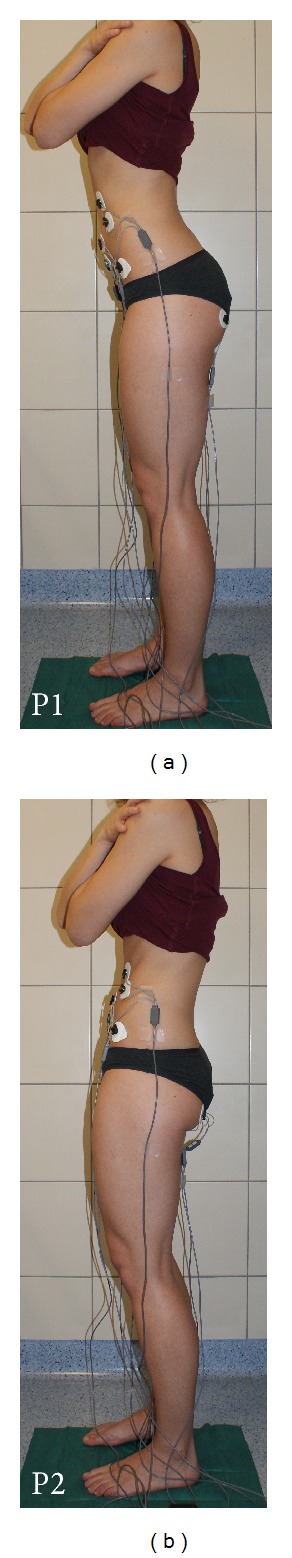
Two positions during the examination (P1—active, the maximum orientation of the pelvis in anteversion performed by patients; P2—active, the maximum orientation of the pelvis in retroversion performed by patients).

Functional and resting electrical activity was recorded from the PFM (with an electromyographic instrument and vaginal probe) in a standing position when the patients had the pelvis rotated forward (Position 1—P1) and backward (Position 2—P2) around the transverse axis (Figure 1) (primary outcome).

In P1 and P2, the participants made five, 5-second maximal isolated contractions of the PFM (functional sEMG activity) with a 5-second rest (resting sEMG activity) between each contraction. A random integer generator (http://www.random.org/nform.html) was used to randomly select the order of the positions in which the PFM were tested. Subjects were given 60 seconds of rest between trials. Before the measurements, the physiotherapist taught the patients how to accomplish a correct PFM contraction. Some preliminary contractions were effected to check that the probe was in the correct placement and that the contractions were being performed properly.

Electrical activity was bilaterally recorded (with an electromyographic instrument and surface electrodes) from the synergistic muscles of the PFM: lower rectus abdominis (RA), the gluteus maximus (GM), the adductor magnus (AM), and only from the left side of the external oblique (EO) (secondary outcome).

### 3.4. Electromyography

Electromyographic measurements were conducted using the Myosystem 1400 (Noraxon, Scottsdale, Arizona, USA). Technical specifications include the following: analog output gain—x1000 standard (5000 selected units); common mode rejection ratio (CMRR)—min 100 dB at 50–60 Hz; input impedance > 100 MΩ on sEMG channels (isolated to >3000 Volts); outputs—analog ±5 Volts all sEMG channels, digital 12-bit resolution per channel from USB port; inputs—8 sEMG channels at ±10 mV max, 8 sensor channels at ±5 Volts max, and power 100–240 VAC at 50/60 Hz (0.9 A max); sEMG amplifier performance—1 uV sensitivity and <1 uV RMS baseline noise; data acquisition—12-bit resolution 8 channels, and USB update to PC every millisecond; high pass cutoff—10 Hz first order on sEMG channels; low pass cutoff-selectable 500 or 1000 Hz on sEMG channels; and physical—width: 28 × 19.7 cm, height: 10.2 cm, and weight: 1400 g. MyoSystem 1400L components are as follows: MyoSystem 1400L instrument, power cord, EMG active cable, and preamplified electrode lead (one per channel)—one channel: 3 snaps, seven channels: 2 snaps; USB cable.

### 3.5. sEMG Data Analysis

sEMG recordings were analysed using Noraxon MyoResearch XP Master Edition Version 1.07 software (Noraxon, Scottsdale, Arizona, USA). Electromyographic data were bandpass-filtered between 50 and 1000 Hz (FIR filter—finite impulse response filter), rectified and smoothed using 50 ms RMS (root mean square), and were expressed in microvolts (*μ*V).

### 3.6. Probe

**Figure 2:**
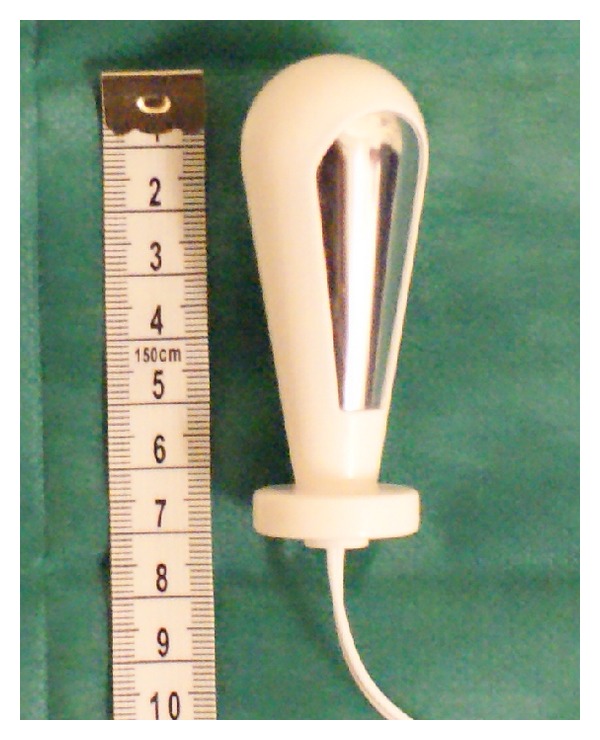
The probe used in the study.

To record sEMG signals from the PFM, a Life-care Vaginal Probe PR-02 (Everyway Medical Instruments Co., Ltd., Taiwan) was used (Figure 2). The probe has a total length of 7.6 cm and a maximal circumference of 2.8 cm. This pear-shaped probe has two longitudinal recording plates (stainless steel, and containing nickel) embedded on the right and left sides and has been reported to record PFM activity with minimal crosstalk during tasks [47–49]. The length of the recording plate is 4.5 cm and the active surface area is 7.68 cm^2^/band. The distance between the two recording plates is 2 cm. The distance of the recording plate to the top of the probe is 0.7 cm and 1.2 cm to the base. This probe is inserted up to the handle at the introitus of the vagina.

The probe was for a single user only. It was cleaned with a pad soaked in surgical spirit, rinsed in clean running water, and dried with a paper towel, before and after each measurement.

### 3.7. Electrodes

The single electrodes are disposable, self-adhesive Ag/AgCl snap electrodes for surface EMG applications only. Diameter of the circular adhesive area is 3.8 cm; diameter of the circular conductive area is 1 cm. Electrodes are hypoallergenic gel and adhesive. The interelectrode spacing between the recording electrodes was 2 cm [50, 51]. The skin was prepared by shaving excess hair and wiping the skin with alcohol (Skinsept pur, Ecolab) to reduce impedance [50–55].

The electrodes were attached parallel to the muscle fibre orientation over the following muscles: RA—the electrodes were below the umbilicus, on the lower rectus abdominis [51, 52]; GM—the electrodes were placed at 50% on a line between the sacral vertebrae and the greater trochanter [56]; AM—the electrodes were positioned midway between the posterior edge of the gracilis and the longitudinal fascial plane, this being between the adductor magnus and the medial hamstrings [57, 58]; EO—the electrodes were just inferior to the 8th rib, in the line of the middle of clavicle and on a 45° angle [48]. The monopolar, reference electrode was placed on the anterior superior iliac spine.

### 3.8. Statistical Analysis

Statistical analyses were performed using Statistica 10 (Stat Soft Inc., USA). For all variables, were calculated, the mean, minimum and maximum values and standard deviation. Differences between the two positions in bioelectrical activity of all muscles were compared using the Wilcoxon signed-rank test. Alpha level was set at 0.05. 

## 4. Results

**Figure 3:**
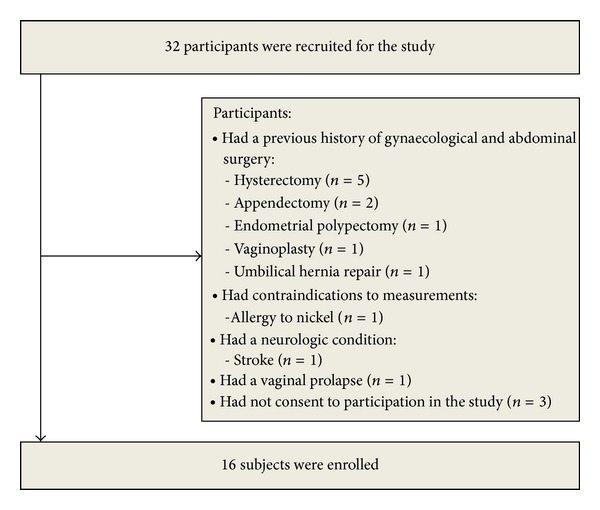
Flow diagram includes detailed information on the excluded participants.

Sixteen women (age: $\bar{x}=63.1$ year, min⁡ = 55 year, max⁡ = 70 year, and SD = 4.07 year) were enrolled in the study. The group characteristics are shown in Table 1. Sixteen were excluded: ten because they had a previous history of gynaecological and abdominal surgery; five because they had not consented to participation in the study; and three because they had a stroke (*n* = 1), were allergic to nickel (*n* = 1), and had vaginal prolapse (*n* = 1) (Figure 3).

### 4.1. Primary Outcomes

**Figure 4:**
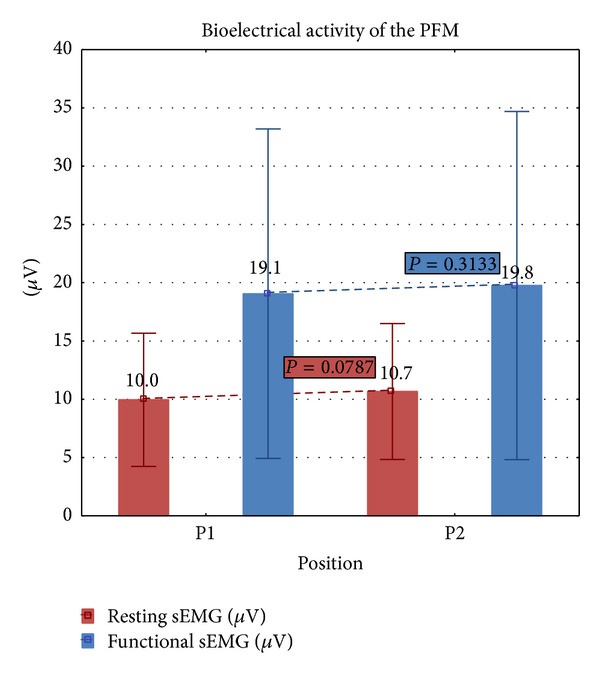
Comparison of resting and functional bioelectrical activity of the PFM in P1 and P2.

No significant differences between P1 and P2 were found in functional and resting sEMG activity of the PFM (Figure 4). In P1, the average resting activity was $\bar{x}=10.0$ *μ*V (min–max: 4.0–22.1 *μ*V; SD = 5.71 *μ*V) and in P2 it was $\bar{x}=10.7$ *μ*V (min–max: 3.9–23.8 *μ*V; SD = 5.83 *μ*V). The average functional activity was $\bar{x}=19.1$ *μ*V (min–max: 5.1–60.9 *μ*V; SD = 14.13 *μ*V) in P1 and $\bar{x}=19.8$ *μ*V (min–max: 5.3–63.7 *μ*V; SD = 14.94 *μ*V) in P2.

### 4.2. Secondary Outcomes

The secondary outcomes are listed in Tables 2 and 3. Significant differences were found between some synergistic muscle activity in orientations P1 and P2.

During resting PFM activity, higher electrical activity in P2 than in P1 has been recorded in the synergistic muscles: GM (left side, *P* = 0.0097) and RA (left side, *P* = 0.0146 and right side, *P* = 0.0386) (Table 2).

During functional PFM activity, higher electrical activity in P2 than in P1 has been recorded in the synergistic muscles: GM (left side, *P* = 0.0061; right side, *P* = 0.0494) and RA (left side, *P* = 0.0113; right side, *P* = 0.0131) (Table 3). Amongst other muscles, no statistically significant differences were registered.

## 5. Discussion

In the present preliminary, prospective study, we evaluated sEMG activity of the PFM in two positions of the pelvis. The results of the present study showed that bioelectrical activity of the PFM does not depend on the orientation of the pelvis. Work by Capson et al. [48] demonstrated the effect of changing the standing lumbopelvic posture on PFM activation amplitude. Amongst other things, they assessed bioelectrical activity of the PFM with sEMG and vaginal probe in three different standing postures (normal lumbopelvic posture, hyperlordosis, and hypolordosis). They did not measure the pelvic tilt, but hypolordotic posture is related to the posterior pelvic tilt. They observed higher resting PFM activity in the hypolordotic posture as compared to the normal and hyperlordotic postures. Chen et al. [59] have reached different conclusions. They examined 39 women with SUI (aged from 38 to 72 years) in order to determine changes in PFM activity triggered by various feet positions. Positioning of the feet was achieved by using a special platform changing the inclination angle of the base on which the examined person was standing. The investigators performed PFM electromyographic measurements by means of an endovaginal probe in three different positions of the feet: plantar flexion, dorsal flexion, and horizontal position. The achieved results showed that during the dorsal flexion of the feet, the anterior pelvic tilt increased and higher PFM activity was observed. Similar studies were carried out by Cerruto et al. [60], who examined 15 women suffering from SUI. Bioelectrical activity of PFM was measured in the standing position with horizontal positioning of the feet and their plantar (5°, 10°, and 15°) and dorsal (5°, 10°, and 15°) flexion. sEMG activity of PFM was significantly higher in the dorsal flexion of the feet, regardless of the flexion angle, in comparison to PFM activity measured for feet in a horizontal position. In the next study, Chen et al. [61] examined 31 healthy women between the ages of 30–56 years. The measurement of PFM tone was performed by means of EMG biofeedback and endovaginal probe. In this study, the PFM tone was measured in a total of 9 positions of feet, active and passive. The highest PFM tone values were achieved in the plantar flexion of the feet with raised upper limbs and were the highest of all values achieved for other positions. In this group, muscle tone, including PFM, could be related to increased intra-abdominal pressure. Bioelectrical PFM stimulation in active positions was higher than in passive positions, probably due to activation of both abdominal wall muscles and the PFM. Such a muscle synergy may probably contribute to more effective PFM training.

We determine, how the values of bioelectrical activity of synergistic muscles were changing during two different orientations of the pelvis. We observed a higher activity of the muscles (GM left side and RA both sides) when the position of the pelvis was backwards. Some studies showed [27, 28, 32–38, 59–61] that the increased activity of these muscles has contributed to increased PFM activity. According to muscle synergy rules, the hip joint muscles located in the vicinity of the PFM have an influence on their activity. A concurrent measurement of the activity of the PFM and the above-mentioned muscles at rest and during contraction has demonstrated the correlation between the examined groups of muscles [27, 28, 32–38]. Soljanik et al. [43] evaluated relations between the levator ani, GM muscle, and the fossa ischioanalis in healthy women. The authors demonstrated cooperation of these structures and revealed synchronous activation of the levator ani and GM in 97% of subjects. The study of Sapsford et al. [62] confirms the increased activity of the PFM and two abdominal muscles, obliquus internus abdominis and obliquus externus abdominis, during various sitting postures. Their highest activity was observed in the very tall unsupported postures. They conclude that unsupported sitting postures require greater pelvic floor muscle activity than the supported ones. Smith et al. [63] conducted a comparison of activity of the PFM and the abdominal muscles between continent and incontinent women in response to a postural perturbation. Women with incontinence demonstrated increased PFM and abdominal obliquus externus sEMG activity compared to continent women. By the authors, in women with more severe symptoms, activity of the abdominal muscles was higher and PFM activity could be insufficient to control continence. Therefore, it is important to assess not only the PFM but also muscles influencing the pelvic floor indirectly.

These studies [32–38, 61–65] have contributed to the verification of the opinion that evaluation and training of synergistic muscles are necessary during the conservative treatment of SUI.

Attention should be given to the practical implications of this study. During the treatment of patients with SUI, exercises of synergistic muscles should also be conducted. The position of the pelvis setting backwards increases the activity of these muscles. The authors believe that the physiotherapy exercise procedure should take into account the position in which the pelvis is set, being a posterior pelvic tilt.

### 5.1. Limitation of the Study

The small number of participants and lack of a control group were limitations of the study. This study will be continued among menopausal women with SUI and without SUI (a control group).

## 6. Conclusion

This preliminary study does not provide initial evidence that pelvic tilt influences PFM activation. Although different activity of synergistic muscles occurs in various orientations of the pelvic tilt, it does not have to affect the sEMG activity of the PFM. Further studies with a control group will contribute to a more accurate assessment of dependence between pelvic tilt and PFM activity.

## Figures and Tables

**Table 1:** Characteristics of group.

		Patients (women, *n* = 16)	
	Range	Mean	SD
Age (year)	55–70	63.1	4.07
Weight (kg)	52–101	75.2	11.35
Height (m)	1.50–1.70	1.61	0.05
ICIQ-SF	1–19	8.3	5.42
MRS	6–22	12.7	4.95

**Table 2:** sEMG activity during resting PFM activity (*μ*V) in P1 and P2.

sEMG activity during resting PFM activity (*μ*V)									
Muscles	P1				P2				*P* value
	Mean	Min	Max	SD	Mean	Min	Max	SD	
Left side									
AM	5.1	2.8	9.9	2.17	6.0	3.4	11.2	2.19	0.1627
GM	5.5	2.8	13.0	3.09	12.8	3.1	68.9	17.75	**0.0097**
RA	4.3	3.3	6.3	0.73	5.1	3.3	9.0	1.53	**0.0146**
EO	13.6	2.5	60.5	13.71	14.1	3.8	59.4	13.26	0.5349

Right side									
AM	5.0	2.7	9.8	2.14	5.5	3.5	11.2	2.24	0.4380
GM	7.7	2.84	22.0	5.86	11.4	2.93	49.0	14.87	0.6417
RA	5.9	3.1	31.0	6.75	6.4	2.7	31.8	6.87	**0.0386**

**Table 3:** sEMG activity during functional PFM activity (*μ*V) in P1 and P2.

sEMG activity during functional PFM activity (*μ*V)									
Muscles	P1				P2				*P* value
	Mean	Min	Max	SD	Mean	Min	Max	SD	
Left side									
AM	7.1	2.9	21.3	5.12	8.2	3.5	18.1	4.79	0.1626
GM	10.9	3.0	69.5	16.20	23.3	3.3	93.8	29.64	**0.0061**
RA	4.6	3.3	6.3	0.85	5.6	3.2	12.4	2.23	**0.0113**
EO	17.4	2.5	70.3	17.65	17.5	3.8	62.8	14.68	0.7564

Right side									
AM	7.3	2.7	17.9	4.68	7.0	3.5	16.8	4.11	0.8361
GM	13.1	3.29	61.6	15.13	19.9	3.36	74.8	24.42	**0.0494**
RA	6.1	3.1	31.1	6.70	6.8	2.7	32.8	7.07	**0.0131**
